# Evaluation of gene expression profiles and pathways underlying postnatal development in mouse sclera

**Published:** 2012-06-02

**Authors:** Wan’E. Lim, Jia Lin Kwan, Liang Kee Goh, Roger W. Beuerman, Veluchamy A. Barathi

**Affiliations:** 1 Singapore Eye Research Institute, Singapore; 2 DUKE-NUS Graduate Medical School, Singapore; 3 Department of Epidemiology & Public Health, Yong Loo Lin School of Medicine, National University, Singapore; 4 Department of Ophthalmology, Yong Loo Lin School of Medicine, National University, Singapore

## Abstract

**Purpose:**

The aim of this study was to identify the genes and pathways underlying the growth of the mouse sclera during postnatal development.

**Methods:**

Total RNA was isolated from each of 30 single mouse sclera (n=30, 6 sclera each from 1-, 2-, 3-, 6-, and 8-week-old mice) and reverse-transcribed into cDNA using a T7-N_6_ primer. The resulting cDNA was fragmented, labeled with biotin, and hybridized to a Mouse Gene 1.0 ST Array. ANOVA analysis was then performed using Partek Genomic Suite 6.5 beta and differentially expressed transcript clusters were filtered based on a selection criterion of ≥2 relative fold change at a false discovery rate of ≤5%. Genes identified as involved in the main biologic processes during postnatal scleral development were further confirmed using qPCR. A possible pathway that contributes to the postnatal development of the sclera was investigated using Ingenuity Pathway Analysis software.

**Results:**

The hierarchical clustering of all time points showed that they did not cluster according to age. The highest number of differentially expressed transcript clusters was found when week 1 and week 2 old scleral tissues were compared. The peroxisome proliferator- activated receptor gamma coactivator 1-alpha (*Ppargc1a*) gene was found to be involved in the networks generated using Ingenuity Pathway Studio (IPA) from the differentially expressed transcript cluster lists of week 2 versus 1, week 3 versus 2, week 6 versus 3, and week 8 versus 6. The gene expression of *Ppargc1a* varied during scleral growth from week 1 to 2, week 2 to 3, week 3 to 6, and week 6 to 8 and was found to interact with a different set of genes at different scleral growth stages. Therefore, this indicated that *Ppargc1a* might play a role in scleral growth during postnatal weeks 1 to 8.

**Conclusions:**

Gene expression of eye diseases should be studied as early as postnatal weeks 1–2 to ensure that any changes in gene expression pattern during disease development are detected. In addition, we propose that *Ppargc1a* might play a role in regulating postnatal scleral development by interacting with a different set of genes at different scleral growth stages.

## Introduction

The sclera is a fibrous connective tissue consisting of 90% collagen and a small percentage of proteoglycans and glycoproteins. Collagen fibrils of the sclera are arranged in layers (lamellae) and are relatively similar to the collagen arrangement of the cornea [1]. The sclera forms the major component of the ocular outer coat and is continuous with the cornea anteriorly [2]. Its main function is to protect the delicate intraocular structures, maintain the optical path for retinal imaging, overcome the intraocular pressure and preserve the ocular dimensions by controlling the size of the eye [3,4].

The mouse eye is not fully developed at birth. After birth, the mouse eye remains closed until approximately 10–14 days, after which eyelid adhesions break down through the processes of apoptosis and necrosis [5]. As the mouse eye develops, adaptive changes occur in response to light exposure and environmental changes.

The corneal stroma and sclera consist of few fibroblasts and large amounts of extracellular matrix (ECM). In general, mRNA levels of ECM and collagen genes have been found to be downregulated in both the adult mouse sclera [6] and cornea [7] when compared with samples from an earlier developmental stage. Also, in a paper by Zhou et al. [6], the transforming growth factor beta 1 (*Tgfβ1*) gene and several collagen genes were significantly downregulated in 8-week-old B6 mice scleras when compared with 3-week-old B6 mice scleras. It was suggested that *Tgfβ1* could possibly act as a signaling molecule in controlling collagen gene expression and modulate ECM during ocular growth. On the other hand, components of ECM have been found to be continually synthesized and accumulated throughout postnatal growth, such as the concentration of scleral proteoglycans, which have been found to increase steadily in humans from childhood to the fourth decade of life [8].

As the sclera plays several pivotal functions in eye, understanding the mechanism of post-natal sclera development will shed light to the pathogenesis of disorders found in sclera as well as other ocular tissues. The mouse genome bears 85% homology to the human genome and has been completely sequenced [9]. Thus, the use of the mouse model has advantages over other animal models, given that we would like to study genetic expression patterns in the mouse sclera tissue to gain an understanding of human postnatal scleral gene expression. Although there have been studies conducted on the developmental growth of the cornea, there are relatively few gene expression studies documenting expression patterns in postnatal mouse sclera. In a previous study on postnatal scleral growth by Zhou et al. [6], total RNA was extracted from pooled mouse sclera (18 samples each from 3- and 8-week-old mice 6, per array chip), converted to biotin labeled cRNA and hybridized to GeneChip® Mouse Genome 430 2.0 Array chips (3′-based expression arrays). The expression of 8 extracellular matrix related genes were then confirmed using real time PCR. In our experiment, we included more time points (namely weeks 1, 2, 3, 6, and 8) to provide a more comprehensive overview of sclera gene expression profiles during postnatal development. Additionally, we used GeneChip® Mouse Gene 1.0 ST Arrays that provide better coverage and a more accurate view of the total transcription at each genomic locus than 3′-based expression array designs do. We also identified pathways underlying the postnatal scleral development using Ingenuity Pathway Analysis software (IPA). Furthermore, RNA from a single sclera was isolated for each gene array to allow the detection of the differences that exist between individual samples. Using high-throughput microarray technology to do gene profiling (genome wide expression analysis) and pathway analysis, we hope to further understand the gene expression profiles and identify a signaling pathway that occurs in sclera during postnatal development.

## Methods

### Animals and tissue collection

Balb/cJ mice (*Mus musculus*) were obtained from the animal holding unit of the National University of Singapore. Approval was obtained from the SingHealth Institute Animal Care and Use Committee (IACUC, AAALAC accredited) and all aspects of this study were performed according to the Association for Research in Vision and Ophthalmology (ARVO) Protocol for animal experimentation. In this study, animals belonging to the same family were used. This was done to minimize changes in gene expression patterns due to differences in genetic background. A single sclera was collected from one mouse (n=30, 6 single sclera from 6 individual mice, 6 mice per time point at week 1, 2, 3, 6, and 8) for each microarray. Principal component analysis (PCA analysis) of microarray results showed little variation among the samples from each week. As a single sclera provides insufficient yields of RNA to confirm the microarray results, sclera from 6 additional individual mice were pooled and subsequently converted to cDNA for the expression analysis of a group of genes.

### RNA, target cDNA and microarray chip preparation

Total RNA was isolated from single cryogenically ground mouse sclera (n=30, 6 samples each from 1-, 2-, 3-, 6-, and 8-week-old mice) using the MELT^TM^ Total Nucleic Acid Isolation System (Ambion Inc., Austin, TX) according to the manufacturer’s instructions. RNA concentration and quality was assessed by absorbance at 260 nm and the absorbance ratio of 260/280 using a Nanodrop® ND-1000 Spectrophotometer (Nanodrop Technologies, Wilmington, DE). The extracted RNA was then reverse-transcribed into sense cDNA using a T7-N_6_ primer and labeled with biotin using a Genechip® Whole Transcript Sense Target Labeling Assay (Affrymetrix, Inc., Santa Clara, CA). Biotin-labeled cDNA were then hybridized to a Mouse Gene 1.0 ST Array (Affrymetrix, Inc.) using a Genechip® Hybridzation Kit (Affrymetrix, Inc.). The microarray chips were washed and stained using a Genechip® Hybridzation, Wash and Stain Kit (Affrymetrix, Inc.). Subsequently, the microarray chips were scanned using a Genechip® Scanner 3000 7G (Affrymetrix, Inc.).

### Microarray data analysis

The microarray gene expression data was analyzed using Partek Genomic Suite 6.5 beta software (Partek Inc., Louis, MO). Briefly, the data (cel.files) was imported and normalized using robust multiarray averaging (RMA). The data discussed in this publication has been deposited in NCBI’s Gene Expression Omnibus (GEO) and is accessible through GEO Series accession number GSE28056.

The samples were assessed for batch effects and further normalized by the batch removal tool using scan date as a factor. ANOVA was then performed to identify significant differentially expressed transcript clusters. The lists of differentially expressed transcript clusters between age groups were filtered based on a selection criterion of ≥2 relative fold change at a false discovery rate of ≤5% (the lists of the differentially expressed transcript clusters between age groups can be found in Appendix 1).

Subsequently, the list of differentially expressed transcript clusters for week 2 versus 1, week 3 versus 2, week 6 versus 3, and week 8 versus 6 were selected for Gene Ontology (GO) enrichment (the rest of the GO analysis between age groups can be found in Appendix 2).

Each functional group was assigned with a GO enrichment score that was calculated using a χ^2^ test. A forest plot was also generated to show the percentage of differentially expressed genes that were upregulated and downregulated for each functional group.

### RNA extraction and quantitative real time PCR (qPCR)

We used qPCR to confirm the expression of genes from the processes that were significantly involved in scleral growth from postnatal week 1 to 2, week 2 to 3, week 3 to 6, and week 6 to 8 based on our GO analysis. In addition, we confirmed that collagen genes (*Col*), epidermal growth factor receptor (*Egfr*), fibrillin 2 (*Fbn2*), Fyn proto-oncogene (*Fyn*), and insulin-like growth factor 2 (*Igf2*) in 1-, 2-, 3-, 6-, and 8-week-old sclera were differentially expressed at all time points.

Total RNA was then isolated from the pooled cryogenically-ground mouse sclera (n=60, six pooled sclera, each from one-, two-, three-, six-, and eight-week-old mice; this was repeated with two independent batches) using TRIzol Reagent (Invitrogen, Carlsbad, CA) according to the manufacturer’s instructions, with some modifications. After homogenization, 0.2 ml of chloroform was added and vortexed for 3 min. The mixture was centrifuged at full speed and the aqueous phase was collected. Isopropanol (0.5 ml of 100%) and 2 μl of glycogen (5 mg/ml; Ambion Inc.) was added and centrifuged at full speed. Last, the RNA was further purified and DNase treated using an RNeasy® Mini Kit (Qiagen GmbH, Hilden, Germany). The RNA concentration and quality were assessed as before.

Purified RNA (500 ng) was reverse-transcribed into cDNA using random primers and reagents from an iScript^™^ select cDNA synthesis kit (Bio-rad Laboratories, Hercules, CA). The primers (see Appendix 3: Gene accession number and qPCR primer sequences) were designed using a ProbeFinder 2.45 (Roche Applied Science, Indianapolis, IN). qPCR was performed using a Lightcycler 480 Probe Master (Roche Applied Science) according to the manufacturer’s instructions. The reaction was run in a Lightcycler 480 (Roche Applied Science) for 45 cycles under the following conditions: 95 °C for 10 s, 56 °C for 10 s, and 72 °C for 30 s.

qPCR data was analyzed using the comparative C_t_ (ΔΔC_t_) method as previously mentioned [10]. Briefly, the average of target gene C_t_ was subtracted by the corresponding average *18S rRNA* C_t_ value to obtain a ΔC_t_ value. Subsequently, the ΔC_t_ value was subtracted by the ΔC_t_ calibrator to obtain the ΔΔ C_t_ value. In this study, ΔC_t_ values of target genes in week 1 were used as a ΔC_t_ calibrator to determine the relative fold change in target genes between weeks 1, 2, 3, 6, and 8. A total of two sets of cDNA were synthesized from two separate batches of mice for running qPCR, and a Student’s *t*-test was performed to determine the significance of the relative fold change.

### Pathway analysis

Ingenuity Pathway software analysis (IPA; Ingenuity® Systems, Redwood City, CA) was used to generate several networks using differentially expressed transcript cluster lists for week 2 versus 1, week 3 versus 2, week 6 versus 3, and week 8 versus 6. Similar significant networks (calculated by Fisher’s exact test; p<0.05) were found in the scleral growth from week 1 to 2, week 2 to 3, week 3 to 6, and week 6 to 8.

## Results

### Significant difference between various age groups

**Figure 1:**
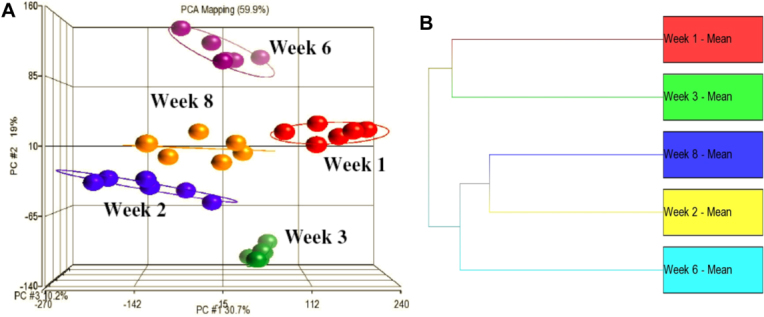
Gene expression data analysis. A PCA plot of all samples was generated using Partek Genomic Suite 6.5 beta to assess the variability of microarray data (Color for scan date and shapes for time points). It was shown that the individual scleral samples from each time point were more related to each other than samples from other time points, demonstrating that there were significant differences in the gene expressions between the various age groups (**A**). Furthermore, the mean of all time points was hierarchically clustered, and it was demonstrated they did not cluster according to age (**B**).

All samples were assessed for batch effects and normalized by the batch removal tool using scan date as a factor. The variability between the age groups was evaluated using a PCA plot (Figure 1A). It was shown that the individual scleral samples from each time point were more related to each other than samples from other time points, demonstrating that there were significant differences in the gene expressions between the various age groups. ANOVA was performed to identify significant differentially expressed genes. The lists of differentially expressed transcript clusters between age groups were generated based on a selection criterion of ≥2 relative fold change at a false discovery rate of ≤5% (Table 1). Unexpectedly, the hierarchical clustering of all time points showed that they did not cluster according to age (Figure 1B). Week 2 versus 1 had the highest number of differentially expressed transcript clusters when the gene expression profiles from week 2, 3, 6, and 8 were compared to week 1. Also, postnatal week 2 versus 1 had the highest number of differentially expressed transcript clusters among all of the possible age comparisons. This suggests that the majority of gene expression changes during scleral postnatal development occurred between postnatal week 1 and 2. Our microarray data showed that week 8 versus 2 had the least number of differentially expressed transcript clusters, indicating that the gene expression profiles in the 2-week-old sclera were most related to the 8-week-old sclera as compared to other weeks. Since mouse eye development starts to reach a plateau from postnatal week 8 onwards, we considered week 8-old sclera to be the same as adult sclera. Therefore, it was surprising that the gene expression profiles of 2-week-old sclera were quite similar to adult sclera. However, there were still 749 transcript clusters found to be differentially expressed between the week 2 and week 8 sclera.

**Table 1:** Differentially expressed transcript clusters found among the age groups.

**Comparison**	Differentially expressed transcript clusters*
Week 2 versus 1	4124
Week 3 versus 1	2538
Week 6 versus 1	2172
Week 8 versus 1	2554
Week 3 versus 2	1530
Week 6 versus 2	2400
Week 8 versus 2	749
Week 6 versus 3	2726
Week 8 versus 3	1809
Week 8 versus 6	1332

*>2 relative fold change (p<0.05).

### Gene ontology (GO) analysis of postnatal week 1 to 2

**Figure 2:**
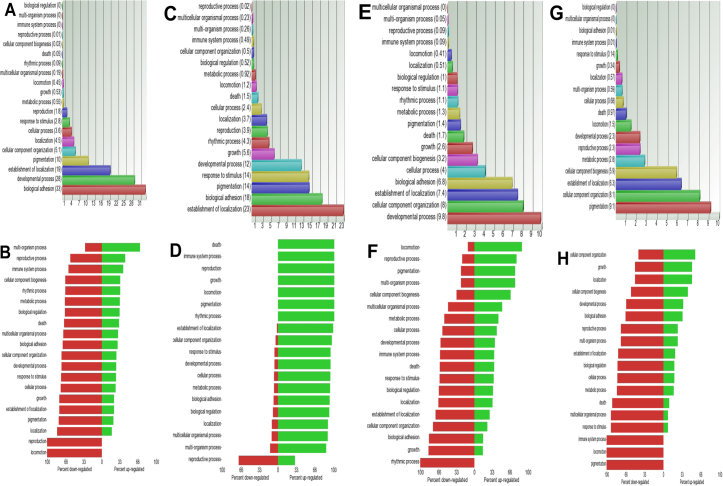
GO analysis of sclera postnatal development. GO analysis was used to identify that the main biologic processes that were involved in scleral growth from postnatal week 1 to 2 (**A**), week 2 to 3 (**C**), week 3 to 6 (**E**), and week 6 to 8 (**G**), respectively. Furthermore, forest plots were generated to analyze the percentage of genes that were either upregulated or down-regulated in each of the biologic processes that was involved in scleral growth from postnatal week 1 to 2 (**B**), week 2 to 3 (**D**), week 3 to 6 (**F**), and week 6 to 8 (**H**), respectively.

GO analysis was used to identify the main biologic processes that were involved in scleral growth from postnatal week 1 and week 2 sclera (Figure 2A). In general, biologic adhesion, developmental process, establishment of localization and pigmentation were the main biologic processes involved. Specifically, the most significant functions that were involved in week 1–2 scleral development were visual perception, calcium ion binding, homophilic cell adhesion, cell-matrix adhesion, nervous system development, eye development, cation (sodium and calcium ions) transport and neurotransmitter transport. The expressions of genes in these processes were mostly downregulated in week 2 sclera when compared to week 1 sclera (Figure 2B).

### GO analysis of postnatal week 2 to 3

Establishment of localization, biologic adhesion, pigmentation, response to stimulus, and developmental process were the main biologic processes involved in scleral growth from postnatal week 2 to 3 (Figure 2C). In particular, the most significant functions that were involved in scleral growth from postnatal week 2 to 3 were visual perception, polysaccharide binding, extracellular matrix binding, neurotransmitter transport, cation (sodium, calcium and potassium) transport, cell-matrix adhesion, homophilic cell adhesion, phototransduction, detection of light stimulus, nervous system development and melanocyte differentiation. Their gene expressions were significantly upregulated in scleral growth from postnatal week 2 to 3 (Figure 2D).

### GO analysis of postnatal week 3 to 6

Generally, developmental process, cellular component organization, establishment of localization and biologic adhesion were the main biologic processes that were involved in scleral growth from postnatal week 3 to 6 (Figure 2E). Particularly, the most significant functions that were involved in scleral development from postnatal week 3 to 6 were muscle system process, muscle organ development, angiogenesis, actin binding, nucleosome assembly, collagen fibril organization, oxygen transport, chloride ion transport, cell-matrix adhesion and leukocyte adhesion. The expressions of genes that were involved in angiogenesis, actin binding, nucleosome assembly, collagen fibril organization, oxygen transport, chloride ion transport, cell-matrix adhesion, and leukocyte adhesion were mostly downregulated, whereas the expressions of genes that were involved in muscle organ development and muscle system process were mostly upregulated in week 6 sclera when compared to week 3 sclera (Figure 2F).

### GO analysis of postnatal week 6 to 8

Pigmentation, cellular component organization, establishment of localization (mainly bicarbonate, chloride ion, and sulfate transport) and cellular component biogenesis were the main biologic processes that were involved in scleral growth from postnatal week 6 to 8 (Figure 2G). Particularly, the most significant functions that were involved in scleral development from postnatal week 6 to 8 were nucleosome assembly, protein-DNA complex assembly, macromolecular complex assembly, melanin biosynthetic process, bicarbonate transport and ribosome biogenesis. The expressions of genes that were involved in nucleosome assembly, protein-DNA complex assembly and macromolecular complex assembly were mostly upregulated, whereas the expressions of genes that were involved in melanin biosynthetic process, bicarbonate transport and ribosome biogenesis were mostly downregulated in week 8 sclera when compared to week 6 sclera (Figure 2H).

### Gene expression of common reference genes

**Figure 3:**
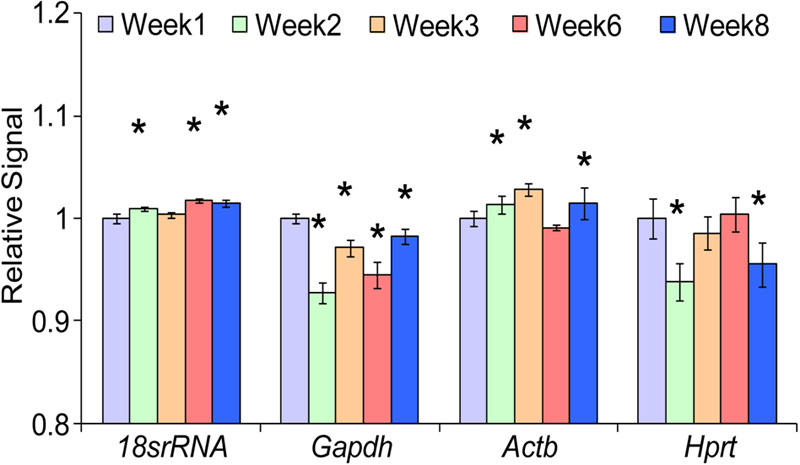
Gene expression of 4 housekeeping genes from microarray data. Mean relative signal (n=6) and standard deviation of 4 housekeeping genes were calculated and plotted to compare their gene expression from week 1, 2, 3, 6, and 8. ANOVA analysis was performed to compare the gene expression of different weeks against week 1 gene expression. * represents p<0.05. It was shown that there were some significant differences in the gene expression of glyceraldehyde-3-phosphate dehydrogenase (*Gapdh*), beta actin (*Actb*), hypoxanthine guanine phosphoribosyl transferase (*Hprt*), and 18S rRNA(*18S rRNA*) when week 2, 3, 6, and 8 were compared with week 1.

While the eye undergoes postnatal development, the gene expression of common reference genes may alter [11]. Therefore, we examined the expressions of some common reference genes to identify a suitable internal control for qPCR normalization. Microarray data showed that there were some significant differences in the gene expression of glyceraldehyde-3-phosphate dehydrogenase (*Gapdh*), beta actin, (*Actb*), hypoxanthine guanine phosphoribosyl transferase (*Hprt*), and 18S rRNA (*18S rRNA*) when week 2, 3, 6, and 8 were compared with week 1 (Figure 3). Nevertheless, qPCR analysis results showed that *18S rRNA* had the lowest standard deviation when its gene expression was compared among 1-, 2-, 3-, 6-, and 8-week-old mice (Table 2). Therefore, *18S rRNA* was subsequently used as a reference gene for normalization of the qPCR data in this study.

**Table 2:** Gene expression of housekeeping genes based on C_t_ value.

**Genes**	**Average C_t_^1^ calue among week 1, 2, 3, 6, and 8**	**Standard deviation**
*18s rRNA*	10.0	0.37
*Gapdh*	22.6	0.72
*Hprt*	27.0	0.63
*Actb*	20.1	0.66

^1^A higher Ct value would indicate a lower level of gene expression.

### Gene expression validation

We used quantitative real-time PCR (qPCR) to confirm the expression of genes from the processes that were significantly involved in scleral growth from postnatal week 1 to 2, week 2 to 3, week 3 to 6, and week 6 to 8, based on GO analysis (Table 3 and Appendix 4). For most of the genes analyzed with qPCR, the fold change regulation direction was similar to the microarray data, but for genes *Tgfβ2*, keratin 17 (*Krt 17*), and histone cluster 1, H2bc (*Hist1h2bc*), a relatively low fold change of less than 1.4 was reported. As the fold change was relatively low, we are unable to confirm the result obtained from the microarray data.

**Table 3:** Comparison of relative fold change of genes from processes that were significantly involved in scleral growth.

**Comparison**	**Genes**	**GO term**	**Microarray (fold change)**	**qPCR (relative fold change)**
Week 2 vs 1	*Col3a1*	collagen fibril organization	−2.7	−1.5
	*Tgfβ 2*	eye development	−2.6	−1.3
	*Neurod1*	eye development	−26.5	−15.4
Week 3 vs 2	*Ctgf*	calcium ion transport into cytosol	4.2	4.3
	*Tgfβ 1*	Regualtion of sodium ion transport	2.0	1.6
	*Dsg1a*	homophilic cell adhesion	2.7	1.6
Week 6 vs 3	*Ttn*	cardiac muscle tissue morpogenesis	26.5	2.0
	*Myo 18b*	cardiac muscle fiber development	4.1	1.4
	*Col 3a1*	collagen fibril organization	−2.1	−2.1
Week 8 vs 6	*Myo 5a*	pigmentation	−2.1	−1.4
	*Krt 17*	intermediate filament cytoskeleton organization	3.1	1.2
	*Hist1h2bc*	nucleosome assembly	2.8	1.2

On the other hand, relative fold change results obtained from qPCR analysis and microarray for some genes were relatively different. This was observed for genes neurogenic differentiation 1 (*Neurod1*) and titin (*Ttn*). This could be due to differences in the normalization methods used between the two different techniques. A global normalization technique was employed when analyzing the microarray data, but this is not possible when doing qPCR analysis. Also, in a study by Etienne et al., an increase in distance between the PCR primers and microarray probes for a given gene resulted in a decrease in the correlation of results between the two different methods [12]. It is thus suggested that primers be designed as close as possible to the distance amplified by the microarray probes.

**Figure 4:**
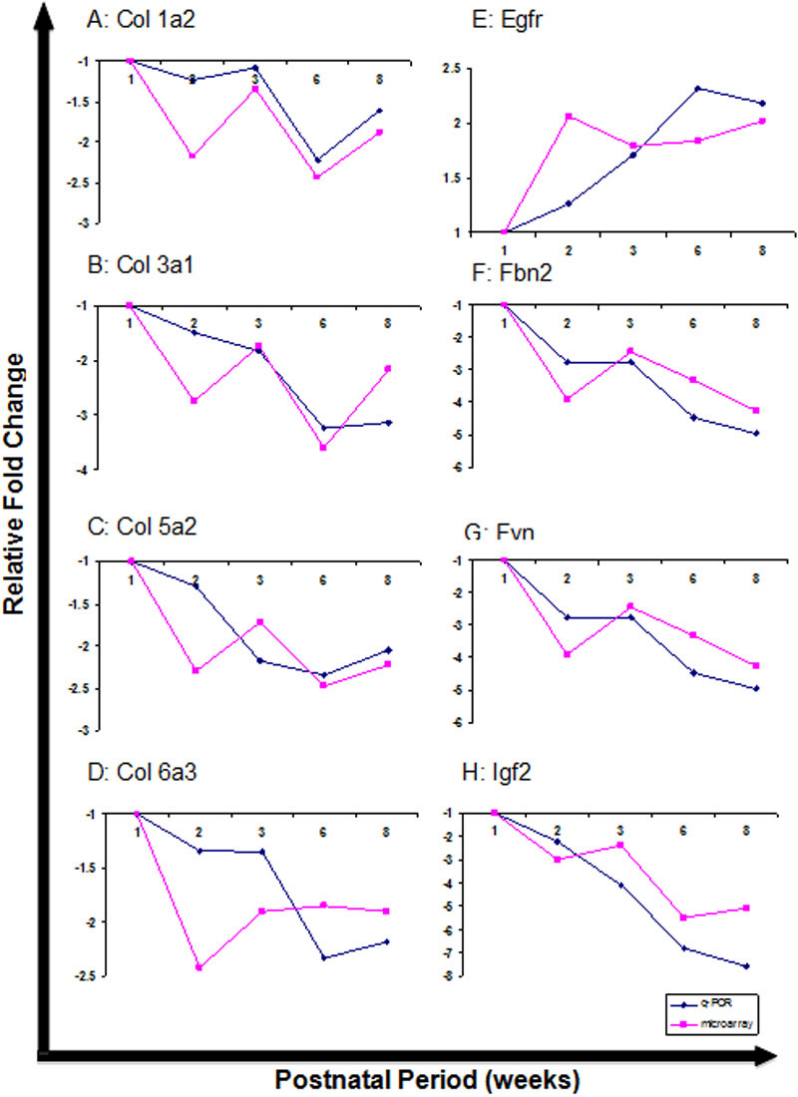
qPCR analysis of collagen genes, *Egfr*, *Fbn2*, *Fyn*, and *Igf2*. The gene expression profiles of *Col-1a2*, *-3a1*, *-5a2*, *-6a3*, and *Egfr*, *Fbn2*, *Fyn*, and *Igf2* in week 1, 2, 3, 6, and 8 were compared the microarray data. It was demonstrated that the expression profiles of these genes obtained from the microarray data showed a similar gene regulation direction, as compared to the expression profiles obtained from qPCR.

Also, we compared the gene expression of *Col-1a2* (collagen, type I, alpha 2), -*3a1* (collagen, type III, alpha 1), -*5a2* (collagen, type V, alpha 2), -*6a3* (collagen, type VI, alpha 3), epidermal growth factor receptor (*Egfr*), fibrillin 2 (*Fbn2*), Fyn proto-oncogene (*Fyn*), and insulin-like growth factor 2 (*Igf2*) in one-, two-, three-, six-, and eight-week-old sclera in both the qPCR and microarray experiments (Figure 4). The fold changes in the gene expressions obtained from the microarray results were higher than the relative fold changes obtained from the qPCR results in some cases. This might be due to the difference in the detection methods and probe design between the microarray chips and qPCR. Each gene was represented by approximately 27 probes that spread across the full length of the gene on the GeneChip® Mouse Gene 1.0 ST Array, and thus the final fold change obtained from the microarray was an average expression change of these probe regions. In contrast, the relative fold change obtained from qPCR represented the change in expression of a selected gene region. Nevertheless, the gene expression profiles obtained from the microarray data showed a similar gene regulation direction as compared to the gene expression profiles obtained from qPCR.

### Ingenuity pathway analysis (IPA)

We used IPA to determine similar significant networks found between scleral growth from week 1 to 2, week 2 to 3, week 3 to 6 and week 6 to 8. It was found that peroxisome proliferative activated receptor, gamma, coactivator 1a (*Ppargc1a*) was involved in the networks generated from the lists of differentially expressed transcript clusters for week 2 versus 1, week 3 versus 2, week 6 versus 3, and week 8 versus 6. This indicated that *Ppargc1a* might play a role in regulating scleral growth from postnatal week 1 to 8, as the gene expression of *Ppargc1a* varied significantly during sclera growth from week 1 to 2, week 2 to 3, week 3 to 6, and week 6 to 8 (Table 4). Furthermore, *Ppargc1a* sometimes interacted with a different set of genes at different scleral growth stages. We also looked into the expression of *Ppargc1a* using qPCR. However, the fold change values obtained from qPCR analysis were lower as compared to the fold change values that were obtained from the microarray data. Particularly, the fold change comparison for week 1 versus 2 and week 3 versus 6 were −1.1 and +1.1 respectively. On the other hand, week 2 versus 3 comparison as well as week 6 versus 8 comparison of *Ppargc1a* expression showed a similar gene regulation direction, as compared to the gene expression profile obtained from the microarray.

**Table 4:** *Ppargc1a* gene expression during sclera postnatal development.

**Scleral growth**	**Microarray fold change**	**qPCR relative fold change**
Week 1 to 2 (Week 2 vs 1)	−6.7	−1.1
Week 2 to 3 (Week 3 vs 2)	2.7	2.5
Week 3 to 6 (Week 6 vs 3)	2.1	1.1
Week 6 to 8 (Week 8 vs 6)	−3.2	−1.8

**Figure 5:**
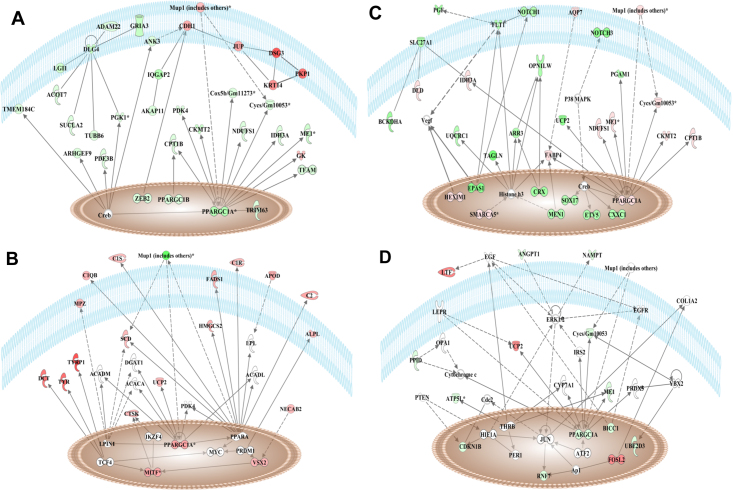
Identification of network with common gene interactions involved in postnatal sclera development using IPA. IPA was used to generate several networks using differentially expressed transcript cluster list from week 2 versus 1 (**A**), week 3 versus 2 (**B**), week 6 versus 3 (**C**), and week 8 versus 6 (**D**). The network from each differentially expressed transcript cluster list with common gene interactions (calculated by Fisher’s exact test; p<0.05) was identified. *Ppargc1a* was the only common gene that was involved in the networks generated from differentially expressed transcript cluster list of week 2 versus 1, week 3 versus 2, week 6 versus 3, and week 8 versus 6, respectively. Red color represents the upregulated genes and green color represents the down-regulated genes from micro array analysis. Intensity of the color indicates the gene expression level.

As depicted in the associated network generated by IPA (Figure 5A), the gene expressions of *Ppargc1a,* malic enzyme 1, NADP (+)-dependent, cytosolic (*Me1*), cytochrome c, somatic (*Cycs*), NADH dehydrogenase (ubiquinone) Fe-S protein 1(*Ndufs1*), creatine kinase, mitochondrial 2 (*Ckmt2*), and carnitine palmitoyltransferase 1b, muscle (*Cpt1b*) were downregulated during scleral growth from postnatal week 1 to 2. In addition, other downstream players such as peroxisome proliferative activated receptor, gamma, coactivator 1b, and zinc finger E-box binding homeobox 2 were also significantly downregulated during scleral growth from postnatal week 1 to 2.

During scleral growth from postnatal week 2 to 3 (Figure 5B), the gene expression of *Ppargc1a* and uncoupling protein 2 (mitochondrial, proton carrier) were upregulated. Furthermore, downstream players such as microphthalmia-associated transcription factor and visual system homeobox 2 were also upregulated during scleral growth from postnatal week 2 to 3.

The network associated with scleral growth from postnatal week 3 to 6 (Figure 5C) showed that *Ppargc1a* was further upregulated from postnatal week 3 to 6. In addition, the gene expressions of *Me1*, *Cycs*, *Ckmt2*, *Cpt1b*, and *Ndufs1* were also upregulated from postnatal week 3 to 6. Furthermore, hexamethylene bis-acetamide inducible 1 gene expression was upregulated while the gene expressions of placental growth factor and Fms-like tyrosine kinase-1 were downregulated during scleral growth from postnatal week 3 to 6.

Based on the network generated by IPA (Figure 5D), the gene expressions of *Ppargc1a*, *Cycs*, and *Me1* were downregulated during scleral growth from postnatal week 6 to 8.

## Discussion

### Differential gene expression patterns detected as early as week 1

Many gene expression studies on eye diseases have been performed using adult mice (≥6 weeks old). However, we have illustrated that changes in gene expression levels in the mouse eye can occur as early as postnatal week 1. Our microarray data showed that the week 2 versus 1 comparison generated the highest number of differentially expressed genes when all weeks were compared, indicating that most of the gene expression change occur during the eye opening process that occurs around postnatal day 12–14. Indeed, Barathi et al. [13,14] had reported that the growth rate of mouse and rabbit eye axial length was the highest from week 1 to 2. Hence, we propose that the gene expression profiles of eye diseases should be studied as early as postnatal week 1 to ensure that all altered genetic changes during disease development are detected.

### Differential gene expression patterns were not age dependent

Previously, Dorrell et al. [15] examined the global gene expression of the developing postnatal mouse retina. The hierarchical clustering of all time points (postnatal day 0, P4, P8, P10, P12, P14, P21, and P42) showed that they did cluster according to age during retinal postnatal development. However, our results showed that changes in gene expression at various time points (postnatal week 1, 2, 3, 6, and 8) during scleral postnatal development was not dependent on age. This variation might be caused by the differences in cell type and function between the sclera and the retina. The retina contains several neuronal types of cells and is mainly responsible for the processing of light signals through phototransduction. On the other hand, the sclera mainly contains fibroblasts that produce and maintain the extracellular matrix that gives the sclera its resilience. Furthermore, these discrepancies in the results might be due to the difference in the type of microarray chips used. We used GeneChip® Mouse Gene 1.0 ST Arrays (whole transcript-based expression array design), whereas Dorrell et al. used the Murine Genome U74v2 genechip (3′-based expression array design) [15]. In general, the whole transcript-based expression array design provides better coverage and a more accurate view of the total transcription at each genomic locus than the 3′-based expression array design does. Therefore, the difference in the array design might also cause slight variations in the results.

### Expression of collagen genes in postnatal mouse scleral

qPCR analysis on postnatal scleral samples revealed a downregulation of collagen gene expression with the increase in postnatal sclera age. This result correlates with the microarray data. One study reported that mRNA levels of collagen (*Col*) I, III, and VI were the highest on postnatal day 5 as compared to postnatal day 20, and at 4, 9, and 15 months [16]. In this study, it was demonstrated that although the mRNA levels of *Col I, III*, and *VI* decreased with increasing age, their proteins were widely distributed in the aging eyes, suggesting a low turnover rate of the collagens [16]. Furthermore, Lee and Davidson [17] reported that the half-life of collagens was longer in older rabbit eyes, and thus collagen synthesis might be reduced in older age. Our results correlated with these studies and showed that the mRNA levels of *Col-1a2*, *-3a1*, *-5a2*, and -*6a3* were the highest in week 1 as compared with week 2, 3, 6, and 8. Therefore, this suggests that the mRNA levels of these collagens in sclera might reduce with increasing age, due to the increasing half-life of their protein.

### Expression of *Igf2* in postnatal mouse sclera

Kane et al. [18] previously reported that the gene expressions of *Igf2* were also downregulated in postnatal day 60 bovine keratocytes as compared to postnatal day 4. Furthermore, *Igf2* were also previously demonstrated to promote synthesis of collagen type I [19]. In agreement with these findings, our results also showed that the gene expression of both *Igf2* and *Col-1a2* were downregulated from week 1 to 8. Therefore, we speculate that the downregulation of the *Igf2* gene might lead to a decrease in mRNA levels of *Col-1a2* in scleral postnatal development from week 1 to 8.

### Expression of *Fbn2* in postnatal mouse sclera

The expression of *Fbn2* in sclera was found to be downregulated with an increase in postnatal age (Figure 4). The results correlated with a microarray study on mouse conjunctiva by Gupta et al. [20], whereby both *Igf2* and *Fbn2* were reported to be among the most decreased transcripts when comparing postnatal day 20 and 9 in conjunctival tissue. In addition, in a paper by Jordan et al. [21], *Fbn2* expression was described to be relatively robust during early fetal development, but expression declined to low levels in postnatal tissue, suggesting it might regulate elastic fiber assembly. *Fbn2* may also act as a mediator for growth signaling by interacting with latent transforming growth factor beta binding protein 3 (*Ltbp-3*) and latent transforming growth factor beta binding protein 4 (*Ltbp-4*) to regulate the targeting of transforming growth factor β (*Tgf-β*) in the ECM [22].

### Expression of *Egfr* in postnatal mouse sclera

Growth factors promote the proliferation of sclera fibroblasts, and thus are important to the growth of the sclera. Hackel et al. had previously reported that *Egf* was involved in ECM production [23]. In our study, *Egfr* gene expression increased progressively from week 1 to 8, and the highest expression was reported in week 2, although the highest expression for qPCR analysis was reported to be at week 6. *Egfr* is an important gene whose key role includes regulating the proliferation of various cell types [24]. Thus, *Egfr* might play a role in promoting scleral fibroblast growth, and hence the overall ocular growth, from week 1 to 8. In addition, our study further added to the importance of *Egfr* in postnatal scleral development whereby the lack of *Egfr* in mice proved embryonically lethal [24].

### Expression of *Fyn* in postnatal mouse sclera

The expression of *Fyn* fluctuated during development, but the expression was downregulated twofold at the end of eight weeks as compared to expression levels in week 1. In a paper by Su et al. [25], fibroblasts that were inactivated for receptor protein tyrosine phoshatase-α caused impairment in the tyrosine kinase activity of both *c-Scr* and *Fyn*. As a result, deficiencies in integrin-mediated signaling responses were exhibited. This implies that *Fyn* gene had a role in regulating integrin signaling, and would be crucial for cell growth and proliferation, considering that integrin mediates the attachment of cell to the tissue surrounding it [25].

### Expression of *Ppargc1a* in postnatal mouse sclera

*Ppargc1a* is a multifunctional transcriptional coactivator and has the ability to interact with different transcription factors in a tissue-specific manner. It plays a significant role in the regulation of several processes, such as mitochondrial biogenesis, adaptive thermogenesis, gluconeogenesis, fatty acid β-oxidation, fiber-type switching and heart development [26-28]. In this study, we suggest that *Ppargc1a* might play a role in scleral development from postnatal week 1 to 8 using IPA. Interestingly, the gene expression of *Ppargc1a* varied significantly during scleral growth from week 1 to 8. Furthermore, *Ppargc1a* interacted with a different set of genes at different scleral growth stages. Previously, it had been reported that human sclera shared common gene expression profiles with cartilage and maintained chondrogenic potential [29]. Indeed, Tsai et al. identified a group of multipotent mesenchymal stem cells residing in sclera and had the ability to differentiate to adipogenic, chondrogenic and neurogenic lineages [30]. As *Ppargc1a* had been shown to act as a coactivator for SRY-related high mobility group-Box gene 9 and to regulate chondrogenesis [31], we propose that *Ppargc1a* might also act as a regulator for scleral postnatal development. Furthermore, *Ppargc1a* might regulate development by interacting with a different set of genes at different scleral growth stages.

Relative fold change values obtained from qPCR analysis of *Ppargc1a* was significantly lower than values obtained from microarray. We are able to confirm the expression of *Ppargc1a* in comparing week 2 versus 3 as well as week 6 versus 8. However, we were unable to confirm *Ppagrc1a* expression for week 2 versus 1 and week 3 versus 6 comparisons. In particular, the fold change in week 2 versus 1 was reduced from −6.7 to −1.1, and week 6 versus 3 was reduced from 2.1 to 1.1. Thus, we are unable to confirm the result for these two weeks, as the fold change values obtained was not significant. This might be due to the difference in detection methods between the microarray chips and qPCR. Nevertheless, the gene expression of *Ppargc1a* still varied during scleral growth from week 1 to 8.

In conclusion, we examined the gene expression profiles of scleral postnatal development from week 1 to 8. The hierarchical clustering of all time points showed that they did not cluster according to age. Despite a short time interval between week 1 and 2, week 2 versus 1 data showed the highest number of differentially expressed transcript clusters when the gene expression profiles in weeks 2, 3, 6, and 8 were compared to week 1. Therefore, we suggest that gene expression of eye diseases should be studied as early as postnatal week 1 to ensure that all altered changes during disease development are detected. In addition, we identified *Ppargc1a* as a possible player in scleral development from postnatal week 1 to 8 using IPA. Hence, we propose that *Ppargc1a* might also act as a regulator for postnatal scleral development by interacting with a different set of genes at different scleral growth stages. Further proteomics investigation will provide a clearer view on the role of the genes that are involved in different stages of postnatal development by confirming their protein expression and localization.

## Appendix 1. Differentially expressed transcript clusters.

To access the data, click or select the words “Appendix 1.” This will initiate the download of a compressed (pdf) archive that contains the file.

## Appendix 2. Gene ontology enrichment analysis of all week comparisons.

To access the data, click or select the words “Appendix 2.” This will initiate the download of a compressed (pdf) archive that contains the file.

## Appendix 3. Gene accession number and qPCR primer sequences.

To access the data, click or select the words “Appendix 3.” This will initiate the download of a compressed (pdf) archive that contains the file.

## Appendix 4. Mean Ct values of all validated genes.

To access the data, click or select the words “Appendix 4.” This will initiate the download of a compressed (pdf) archive that contains the file.
